# Modulation of RNA stability regulates gene expression in two opposite ways: through buffering of RNA levels upon global perturbations and by supporting adapted differential expression

**DOI:** 10.1093/nar/gkac208

**Published:** 2022-04-07

**Authors:** Marie-Line Faucillion, Anna-Mia Johansson, Jan Larsson

**Affiliations:** Department of Molecular Biology, Umeå University, 901 87 Umeå, Sweden; Department of Molecular Biology, Umeå University, 901 87 Umeå, Sweden; Department of Molecular Biology, Umeå University, 901 87 Umeå, Sweden

## Abstract

The steady state levels of RNAs, often referred to as expression levels, result from a well-balanced combination of RNA transcription and decay. Alterations in RNA levels will therefore result from tight regulation of transcription rates, decay rates or both. Here, we explore the role of RNA stability in achieving balanced gene expression and present genome-wide RNA stabilities in *Drosophila melanogaster* male and female cells as well as male cells depleted of proteins essential for dosage compensation. We identify two distinct RNA-stability mediated responses involved in regulation of gene expression. The first of these responds to acute and global changes in transcription and thus counteracts potentially harmful gene mis-expression by shifting the RNA stability in the direction opposite to the transcriptional change. The second response enhances inter-individual differential gene expression by adjusting the RNA stability in the same direction as a transcriptional change. Both mechanisms are global, act on housekeeping as well as non-housekeeping genes and were observed in both flies and mammals. Additionally, we show that, in contrast to mammals, modulation of RNA stability does not detectably contribute to dosage compensation of the sex-chromosomes in *D. melanogaster*.

## INTRODUCTION

The term gene expression is used liberally to refer to anything from the steady state levels of gene products, either mRNA as an intermediate stage or protein as the final effector molecule, to the generation of gene products, thereby describing measures of transcription or translation output. Commonly, the term refers to the steady state levels of RNA. However, the dynamics of gene expression require additional steps that are often overlooked: the decay of the RNAs and the decay of the proteins, since the steady state levels of a molecule are defined by both its production and its decay rate, which are equally important (1). In fact, it was brought forward that the modulation of RNA stability is essential in order to quickly down-regulate otherwise stable RNAs after a change in conditions because such down-regulation cannot be explained solely by a complete transcriptional shut-off (2,3).

The maintenance of balanced gene expression is central for the fitness of an organism, and multiple mechanisms across all levels of gene expression must cooperate and accommodate for changes in gene dosage in order to contribute to the final harmony. In cases of failure, the alternative gene dose will not be passed on to the next generation. The broad definition of dosage compensation englobes the consequences of mechanisms whose purpose is to restore the ‘original expression levels’ following a change in gene dose. This difference in gene dose can be acquired across a long timescale through evolution, as is the case for the heteromorphic sex chromosomes. Alternatively, these changes can occur rapidly following segmental chromosomal duplication or deletion (or even monosomies and trisomies, when these conditions are viable, such as trisomy 21 in humans and monosomy or trisomy of the fourth chromosome in *D. melanogaster*). The latter form of compensation is generally referred to as buffering, and it can also include compensation for gene mis-expression following, for example, single mutations in transcription factors with broad targets (4–6). Our view is that buffering is not expected to be effected through a single mechanism, but rather the result of a set of strategies aiming toward the same goal: minimizing the differences in final gene expression, and can therefore occur both at the RNA level and at the protein level.

Dosage compensation of the sex chromosomes in XX/XY systems aims to rebalance gene expression between the X-chromosome and the autosomes and also between males and females subsequent to the evolution of the sex chromosomes where males have lost one gene dose of most X-linked genes through the degeneration of the Y-chromosome (7,8). In mammals, this is achieved through the random inactivation of one of the two female X-chromosomes (9), potentially accompanied by, a still controversial 2-fold increase in expression from the X-chromosome in both sexes (10–16). Recent analyses, focused on regulation of expression beyond transcript levels in mammals, suggest that higher RNA stability and higher translation rates of the X-chromosome contribute to such chromosome specific increase in expression (17,18).

In *D. melanogaster*, where it is widely accepted that there is a 2-fold increase in RNA levels from the male X-chromosome (19–21), dosage compensation of the sex chromosomes is thought to result from a combination of general buffering effects that act on all monosomic regions and the specific targeting and stimulation of transcription of the male single X-chromosome by the male-specific lethal (MSL) complex (5,21–24). However, it is still not clear whether the increase in transcription is due to increased transcriptional elongation, initiation or a combination of both (25–27). It should be noted that a large proportion of genes have been reported to be dosage compensated without being targeted by the MSL complex (4).

In the current study, we aim to explore the role of RNA stability in establishing balanced genome-wide expression and to provide the community with genome-wide data on RNA stability in *D. melanogaster*. To accomplish this, we determined RNA half-lives for transcripts from 60 to 70% of all genes expressed in *D. melanogaster* male and female cells. Our approach is based on BRIC-seq, a non-destructive method for the estimation of the physiological decay rates of RNAs genome-wide (28). We analyzed differences in RNA half-lives and ribosome densities to determine whether and how differential RNA stability and translational activity are mechanisms involved in maintaining balanced expression.

Here, we uncover two RNA stability mediated responses for the regulation of gene expression. The first is a general response that buffers RNA levels following an induced change in transcription output and acts on all chromosomes by modulating RNA stability in order to counteract both induced decreases and increases in transcription. The second acts on adapted differential expression and enhances, for example cell type specific adapted expression that is established at the transcriptional level. Additionally, we find no evidence of a role for RNA stability in X-chromosome dosage compensation in flies.

## MATERIALS AND METHODS

### Cell lines and cell culture

Schneider’s *Drosophila* line 2, S2 DRSC (male) and Kc167 (female) cell lines were cultivated in Schneider’s medium modified with L-glutamine (Lonza) and supplemented with 10% Fetal Bovine Serum, 100 units/ml penicillin and 100 μg/ml streptomycin. The S2 cells were grown in T-flasks at 25°C and the Kc167 cells were grown in suspension in Erlenmeyer glass flasks in a cabinet at 23°C.

### RNAi treatment of *Drosophila* cells

The DNA templates used to make the dsRNA for *msl2* and *mle* RNAi were obtained by PCR using genomic DNA from wild type Oregon R flies and the following primers:

F^msl2^: TAATACGACTCACTATAGGGAGAGTTGGCTGTGCTGGCTG,

R^msl2^: TAATACGACTCACTATAGGGAGATGTTGGCTCGTCACTGTC,

F^mle^: TAATACGAACTCACTATAGGGGCAACAGGATGGCGAAAAA,

R^mle^: TAATACGACTCACTATAGGGTCTGGGTAGTCTTTCCGCAC.

The DNA template used to make the dsRNA for *yfp* RNAi was obtained by PCR using pEYFP-N1 plasmid DNA (Clontech) and the following primers to introduce the T7 promoter:

F^eYFP^: TAATACGACTCACTATAGGGAGAGGTGAGCAAGGGCGAGGAGCT,

R^eYFP^: TAATACGACTCACTATAGGGAGATCTTGAAGTTCACCTTGATGCCG.

The DNA templates were purified and dsRNA was generated using the T7 RiboMAX Express Large Scale RNA production system (Promega) or the T7 High Yield RNA Synthesis Kit (NEB) according to the manufacturer’s instructions.

### BRIC (Bromouridine Immunoprecipitation Chase)

Our BRIC protocol is based on a protocol from (28) which we have adapted to the two *D. melanogaster* cell lines used. Briefly, all RNAs of a cell are labeled with the uridine analog 5-bromouridine (BrU). Next, the cells are washed and resuspended in fresh media without BrU. From this point (*t* = 0) on, the cells are allowed to continue growing in normal conditions and the RNAs that are degraded will be replaced over time by newly synthesized unlabeled RNAs. Cell samples are collected at multiple time points and the BrU labeled RNAs are separated by immunoprecipitation using an anti-BrU antibody. The BrU labeled RNAs are then sequenced, and the individual RNA half-lives can be deduced from the rate at which transcripts leave the BrU labeled fraction of RNAs.

### RNAi and BrU labeling

A total of 2 × 10^6^ (S2, DRSC) living cells/ml per time point were seeded in 6-well plates or cell culture flasks and incubated for 15–60 min at 25°C to allow the cells to attach to the surface. For the RNAi experiment, 2 × 10^6^ live cells/ml were collected and resuspended in serum-free antibiotic-free medium. About 20 μg dsRNA/ml were added to the cell culture, followed by 30 min incubation at room temperature. Then the cell cultures were diluted 1:1 with medium containing 20% fetal bovine serum, 200 units/ml penicillin and 200 μg/ml streptomycin and incubated at 25°C in flasks/plates for 4 days. The RNAi treatment was repeated as above on the same cells resuspended at 2 × 10^6^ live cells/ml and incubated for 1 more day. For western blot analysis, protein extracts were run on a 10% SDS–PAGE gel and thereafter transferred to a PVDF membrane for 2 h 30 min at 25 V. Primary and secondary antibodies [rabbit anti-MLE (1:10 000) and donkey anti rabbit-HPR (Jackson ImmunoResearch, 1:10 000), mouse anti-tubulin (SIGMA, T5168, 1:10 000) and goat anti mouse-HPR (Thermo Scientific, 1:10 000)] were diluted in 1× PBS, 1% BSA, 0.05% Tween-20. Note that MSL2 was not assessed by western blot due to the poor performance of our MSL2 antibodies in that assay. For the labeling, Bromouridine (BrU) (Sigma or Alfa Aesar) dissolved in culture medium was added to the samples to a final concentration of 10 mM every third hour for 24 h (for the RNAi experiments, dsRNA was also added every third hour to a final concentration of 10 μg/ml).

For labeling the Kc167 cells, cells were resuspended at a concentration of 1 × 10^6^ cells/ml in an Erlenmeyer flask with shaking and incubated for 24 h with a first addition of fresh BrU at a final concentration of 400 μM at the beginning and a second addition of fresh BrU after 12 h.

### Sample collection

After 24 h of BrU labeling, all cells were washed twice with fresh medium (or PBS). Samples of 2 ml for the S2 cells and 10 ml for the Kc167 cells were collected at specific time points (0, 1, 2, 4, 8, 12 and 16 h) by removing the media and resuspending the cells in appropriate amount of TRIzol LS (Ambion Life Technology) for storage at −80°C.

### RNA preparation and immunoprecipitation

RNA was extracted by the alternative protocol provided by the manufacturer of TRIzol LS (Ambion Life Technology) that uses 1-bromo-3-chloropropane (BCP) instead of chloroform. According to the manufacturer, BCP reduces the risk of DNA contamination of RNA samples and is less toxic than chloroform. The RNA was resuspended in 20 μl of RNAse-free water and incubated at 55–60°C for 10–15 min and the concentrations were measured with a NanoDrop (Thermo Scientific). Immunoprecipitation of BrU labeled RNA from S2 cells was done according to the protocol described in (28) while we used a BRIC-kit (MBL) for the Kc167 samples and followed the manufacturer’s protocol.

### Library preparation and sequencing

The sequencing libraries were made with TruSeq RNA Sample Preparation v2 (Illumina) according to the manufacturer’s instructions. The samples were sequenced at different times, all on Illumina sequencing machines (HiSeq2500 or NovaSeq6000) with paired-end sequencing and a read length of 126 and 151 nucleotides respectively. All sequencing data were mapped to *D. melanogaster* genome version 6.33 using STAR version 2.7.0e and default parameters. For each gene and each time point, reads were counted using HTSeq version 0.9.1. The BRIC-seq and RNA-seq data reported in this paper have been deposited in the European Nucleotide Archive with accession number PRJEB15335.

### Half-life calculation

To remove genes expressed at low levels from our dataset, genes with a sum of raw read counts below 10 as well as genes with a cpm (count per million as determined by EdgeR) below 2, for time point *t* = 0, *t* = 1 or *t* = 2 h were filtered out. For genes with rapidly decaying transcripts, the latest time points give very low read counts and the signal is mainly noise, therefore we filtered out all time points with a cpm below 0.5 as well as the period of time coming after a filtered out time point. For the latest time points, in some cases the cpm values increase again due to the fact that the composition is biased toward stable genes. If the increase from one time point to the next is >200%, then the later time point is removed together with all the subsequent time points. In order to correct for biased gene composition at later time points, we calculated and applied normalization factors. To do this, only genes with data for all time points were sequentially fitted to an exponential decay curve using the nls function from the nls2 package in R. Correction coefficients that turn the gene decay curve into a perfect exponential decay curve were collected for each gene and each time point and averaged per time point to form the normalization factors. These factors were applied to the full dataset.

To calculate half-lives, the available time points for each gene were fitted to an exponential decay curve using nls with the formula a*exp(-b*t) and the half-lives were calculated as ln(2)/*b*. The replicates were pooled together by calculating the average if both values were between 0 and 16 h. Half-life values above 16 h or negative indicate that the RNAs are very stable and these genes were arbitrarily given a 16 h half-life. Of the genes classified as expressed, we were able to determine the half-life for 60–70%. For the remaining genes, the decay curve could not be fitted because either its shape was very different from a first order exponential decay or we did not have enough valid time points due to the very rapid decay of some weakly expressed genes.

### GeTMM calculations

GeTMM was chosen to calculate and compare transcript levels because it performs well for both intrasample and intersample comparisons, and the GeTMM values were calculated as presented in (29). Briefly, these values are obtained by inputting TPMs instead of raw read counts into the edgeR normalization method TMM (Trimmed Mean of M-values). In this way, gene length is taken into account in normalization. Genes were defined as expressed, and were thus included, if the transcript level was > 0.2 GeTMM in both our male (S2) and female (Kc167) samples.

### Ribosome density calculations

The RNA-seq and ribo-seq data were generated by (30) and the read counts for coding sequence (CDS) were downloaded from GEO accession numbers GSM2845525 and GSM2845527. TPMs for both experiments were calculated as described in (29) using the sum of non-overlapping CDS as gene length. Genes having a raw read count for the ribo-seq data < 10, a raw read count for the RNA-seq data < 50, a TPM value for the ribo-seq data < 2 or a TPM value for the RNA-seq data < 5 were filtered out. For the remaining genes, the ribosome density was calculated for each gene, as the ratio of the TPM from the ribo-seq experiment over the TPM from the RNA-seq experiment.

### Correlation with poly(A) tail length

The poly(A) tail length dataset was generated by (31) using their method called PAL-seq. Data were retrieved from GEO with the accession number GSM1316798. FlyBase transcript IDs were matched with the corresponding FlyBase gene IDs and merged with our RNA stability data. In our graphs, the mean poly(A) tail length is used.

### Classification into housekeeping genes and non-housekeeping genes

Genes with > 6 as the expression level in all 12 FlyAtlas-specified tissue types (32) were defined as housekeeping genes and genes with > 6 expression levels in 11 or fewer tissue types were defined as non-housekeeping genes.

### Differential expression analysis

For the BRIC experiments, the time point *t* = 0 for each sample was used for differential expression analysis because the BrU labeling procedure may affect gene expression slightly and since we compare differential expression with our calculated half-lives that require BrU labeling, the *t* = 0 time point constitutes a better control than RNA-seq of unlabeled sample. Genes for which the sum of raw counts across all experiments was below 10 were excluded from the analysis. Fold-differences in expression between either *msl2* RNAi or *mle* RNAi and *yfp* RNAi (control) were calculated using the DESeq2 R software package.

### Gene ontology analysis

To identify the biological themes enriched in the long and short half-life RNAs, we performed Gene Ontology (GO) term enrichment analysis for the 20% least stable gene transcripts and the 20% most stable gene transcripts for the two different cell lines. We used the Functional Annotation Tool in the Database for Annotation, Visualization and Integrated Discovery (DAVID) v6.8 (33) to extract the top 5 most significant GO terms enriched for biological process, cellular component and molecular function.

### Definitions

In this study, all data and calculations referring to ‘autosome’ or ‘A’ represent the merged data for the main autosome arms 2L, 2R, 3L and 3R. We exclude chromosome 4 from the autosome group due to its role as an ancestral sex chromosome (34,35). The term ‘transcript levels’ includes all transcripts for each gene. In all figures except the one showing ribo-seq data, ‘RNA length’ is defined as the sum of non-overlapping exons. Throughout the text the term ‘RNA stability’ refers to the stability of the RNAs we could measure, which are polyadenylated RNAs which include mostly mRNAs and lncRNAs.

### Bioinformatics and visualization

All calculations and statistical analysis were performed using R-4.0.3 and the ggplot2 package was used to generate plots. Adobe Illustrator was used to build the figures. The existing BRIC-seq data in UPF1 depleted mammalian cells (36) was kindly provided by Dr Akimitsu and the RNA stability data in lymphoblastoid cell line data (37) was kindly provided by Dr Duan. The half-life values and RNA level values for the biological and technical replicates for each of the seven lymphoblastoid cell line samples were averaged since they had a high degree of correlation in the original study. The data were annotated using the ‘GeneID’ and ‘refseq_mrna’ attribute of BiomaRt. We annotated 10 280 genes among the already filtered genes as being expressed in the original study and the BiomaRt gene biotypes ‘lncRNA’ and ‘protein_coding’. The log2 ratios for the half-life and RNA level values were computed for each pair of samples.

## RESULTS

### Transcript stability is linked to gene function and conserved through evolution

To determine RNA stability genome-wide in *D. melanogaster*, we chose a non-destructive method to minimize the disturbance of the natural physiology of the cell. We adapted our protocol from the BRIC-seq method which was developed for mammalian cells by (28). Briefly, all RNAs are labeled with BrU, the BrU is then removed from the media and samples are collected at different time points. The labeled and non-labeled RNAs are separated by immunoprecipitation and the decline of the proportion of labeled RNAs over time is used to determine decay curves that are used to calculate the half-lives. The dataset we generated consists of genome-wide RNA half-life data for male cells (S2), for female cells (Kc167), and for three RNAi treated male cell samples (S2): *msl2* and *mle* to explore the role of the dosage compensation complex, and *yfp* as an RNAi control sample (Supplementary Table S1). Both the S2 and the Kc167 cell lines originate from embryonic tissues and are among the most commonly used, therefore they were considered to be the most appropriate lines for comparison of expression. The efficiency of the RNAi treatment was confirmed with western blot for MLE and differential expression of RNA-seq for *mle* and *msl2* (Supplementary Figure S1 and Supplementary Table S2). Moreover, the reduction of the average X-chromosome expression in our RNAi experiments was comparable to what has previously been observed both following RNAi-mediated depletion of MSL proteins and also in flies mutant for components in the dosage compensation system (6,38–41). The two replicates for each condition correlate appropriately (Supplementary Figure S2A) and the calculated half-lives were merged. The female cells (Kc167) tolerated less BrU, leading to more noise as compared to our male (S2) samples, and this is likely to explain the lower correlation observed for the female (Kc167) replicates. It should be noted that as a consequence of the complex procedure; including immunoprecipitations, several time points and curve fitting of the data; calculated half-lives are more variable between replicates as compared to standard RNA-seq (42). We therefore base our analysis on comparing gene groups (according to characteristics such as chromosomes, RNA levels, ribosome density and binnings of some of these values) and not individual genes.

**Figure 1.**
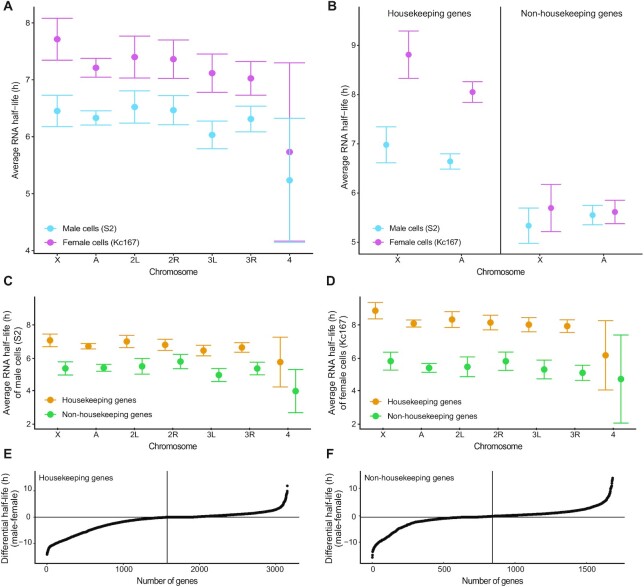
RNA stabilities are regulated and related to gene functions. (**A**) Average RNA half-life per chromosome and for the autosomes for S2 cells (blue) and Kc167 cells (pink). (**B**) Average RNA half-lives for chromosome X and autosomes, for S2 cells (blue) and Kc167 cells (pink), divided into housekeeping and non-housekeeping genes. (**C** and **D**) Differences in RNA half-lives between S2 cells and Kc167 cells for housekeeping genes (**C**) and for non-housekeeping genes (**D**). (**E** and **F**) Differential RNA half-lives between males and females in increasing order. The vertical line represents the median. Genes with RNAs more stable in females are on the left side of the curve and those more stable in males are on the right. All error bars indicate the 95% confidence interval of the mean.

We calculated the RNA half-lives for RNAs from 60 to 70% of all expressed genes (Supplementary Tables S1 and S3) and close to 100% of all housekeeping genes (Supplementary Figure S2B). The transcripts from the female cell line (Kc167) are on average consistently more stable than the transcripts from the male cell line (S2) (Figure 1A and Supplementary Figure S2C). This is in contrast to our previous analysis of human cells, where chromosomal average mRNA half-life in male and female lymphoblastoid cell lines did not reveal any significant difference between the two sexes (18).

It has previously been shown that in mammalian and yeast cells certain categories of genes tend to have either long-lived or short-lived RNAs (28,37,43–46). To assess whether RNA stability is also coupled to gene function in *D. melanogaster*, we analyzed gene ontology (GO) enrichments for genes encoding the 20% most stable RNAs and the 20% least stable RNAs in male (S2) cells and female (Kc167) cells. The results show that RNAs from genes defined by GO terms like translation and mitochondrion are long-lived while genes defined by GO terms such as transcription regulation are enriched in short lived RNAs, in both male cells (S2) and female cells (Kc167) (Table 1 and Supplementary Table S4). An analogous analysis, conducted in mammalian cells by (28), gave similar results. Moreover, (44) found a very good correlation of half-lives ratios between orthologs in mouse and human cell lines. Taken together, this indicates that the relationship between gene function and RNA stability, either classified as stable or unstable has been conserved in the course of evolution.

**Table 1.** Top 5 ranked significantly enriched Gene Ontology Biological Process terms for long and short half-life in S2 and Kc167 cells. The 20% most stable RNAs are classified as long half-life RNAs and the 20% least stable RNAs are classified as short half-life RNAs

GO term	Definition	Number of GO term	Fold enrichment	Adjusted p-value
**S2 long half-life RNAs**				
GO:0002181	Cytoplasmic translation	79	9.105702	8.53E-64
GO:0006120	Mitochondrial electron transport, NADH to ubiquinone	26	8.099496	1.34E-18
GO:0006412	Translation	108	2.071265	1.67E-13
GO:0032543	Mitochondrial translation	30	3.974554	7.85E-11
GO:0015992	Proton transport	15	6.649734	4.41E-09
**S2 short half-life RNAs**				
GO:0022008	Neurogenesis	124	2.592139	5.46E-24
GO:0000462	Maturation of SSU-rRNA from tricistronic rRNA transcript	16	6.299177	3.35E-09
GO:0006351	Transcription, DNA-templated	66	2.0732	1.60E-08
GO:0006261	DNA-dependent DNA replication	11	8.120032	8.93E-08
GO:0016567	Protein ubiquitination	34	2.750497	1.24E-07
**Kc167 long half-life RNAs**				
GO:0002181	Cytoplasmic translation	69	7.757914	3.23E-48
GO:0006120	Mitochondrial electron transport, NADH to ubiquinone	23	6.989112	1.51E-14
GO:0015992	Proton transport	15	6.48655	6.09E-09
GO:0006457	Protein folding	31	3.227259	9.39E-09
GO:0006412	Translation	93	1.73982	1.03E-07
**Kc167 short half-life RNAs**				
GO:0006351	Transcription, DNA-templated	91	2.828125	2.81E-20
GO:0022008	Neurogenesis	97	2.006173	2.41E-11
GO:0006357	Regulation of transcription from RNA polymerase II promoter	40	2.701836	1.30E-08
GO:0006355	Regulation of transcription, DNA-templated	68	1.981571	5.81E-08
GO:0045944	Positive regulation of transcription from RNA polymerase II promoter	46	2.357589	7.44E-08

Next, to check whether transcripts from housekeeping genes were consistently more stable than transcripts from non-housekeeping genes, we calculated and plotted the average RNA half-life per chromosome for these two groups, for both female (Kc167) and male (S2) cells (Figure 1B). The results confirm that RNAs from housekeeping genes are significantly more stable than RNAs from non-housekeeping genes both in male cells (S2) and in female cells (Kc167), overall as well as when analysed chromosome-wise (Figure 1C and D). Additionally, they suggest that the difference in average RNA half-lives between male and female cells is caused mainly by greater stability of housekeeping gene transcripts in the female Kc167 cells (Figure 1B). Still, the Mann–Whitney *U* tests returned significant differences between the sexes for both non-housekeeping genes (*P* = 1.90*10^–8^) and housekeeping genes (*P* = 1.19*10^–11^), indicating significant differences in the distributions. We therefore asked whether RNA stability exhibits gene specificity or cell-type specificity and plotted the gene-wise differences in RNA stability between males and females in increasing order. The curves confirm the statistical test results and show that a large fraction of genes is differentially stable between S2 and Kc167 cells, for both housekeeping (Figure 1E) and non-housekeeping genes (Figure 1F). The distribution is more unbalanced for housekeeping genes; as an example, 553 genes have a >5 h longer half-life in females compared to males whereas only 53 genes have a >5 h longer half-life in males compared to females. As a comparison, among non-housekeeping genes, 171 genes have a >5 h longer half-life in females whereas 82 genes do so in males.

We conclude that RNA stabilities are related to gene functions and the ‘stable’ or ‘unstable’ ranges for RNA stability have been conserved during evolution. However, the observed differential RNA stability of genes between male and female cells suggests that RNA stability is not an entirely intrinsic character based on the RNA sequence but adapts to the cellular and genetic context.

### RNA stability correlates positively with steady state RNA levels and negatively with RNA length while gene function determines the correlation with poly(A) tail length

**Figure 2.**
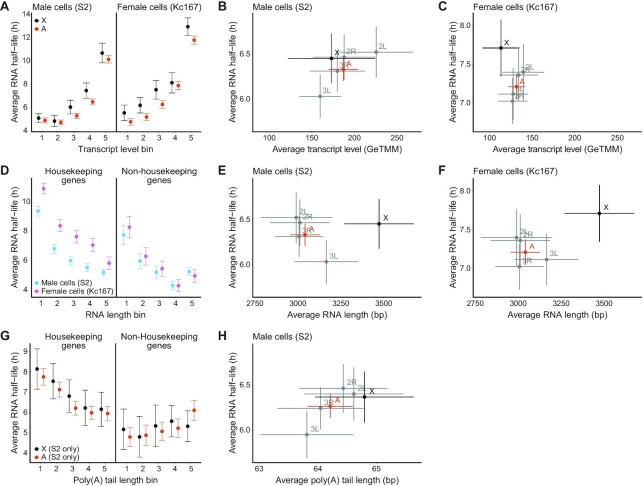
RNA stability correlates positively with steady state transcript levels and negatively with mRNA length; correlations with poly(A) tail length depend on gene function. (**A**) Average RNA half-life for the X-chromosome (black) and the autosomes (orange) for bins with equal numbers of genes and of increasing transcript levels (GeTMM) for S2 cells (left) and Kc167 cells (right). (**B** and **C**) Average transcript levels (GeTMM) per chromosome and for the main autosome arms plotted against their respective average RNA half-life in S2 cells (B) and in Kc167 cells (C). (**D**) Average RNA half-life for S2 cells (blue) and Kc167 cells (pink) for bins of equal numbers of genes and of increasing RNA length (bp) for housekeeping genes (left) and non-housekeeping genes (right). (**E**) Average RNA length (bp) per chromosome and for the main autosome arms plotted against average RNA half-life in S2 cells. The Spearman correlations between RNA length and RNA half-life are: -0.23 (*P* = 1.66*10^–13^) and -0.25 (*P*-value = 5.94*10^–70^) for the X-chromosome and autosomes respectively. (**F**) Average RNA length (bp) per chromosome and for all autosomes plotted against average RNA half-life in Kc167 cells. The Spearman correlations between RNA length and RNA half-life are: -0.29 (*P* = 2.09*10^–21^) and -0.33 (*P* = 2.09*10^–121^) for the X-chromosome and autosomes respectively. (**G**) Average RNA half-life for the X-chromosome (black) and the autosomes (orange) for 5 bins with equal numbers of genes and of increasing poly(A) tail length for housekeeping genes (left) and non-housekeeping genes (right). The Spearman correlation between poly(A) tail length and RNA half-life for housekeeping genes is -0.14 (*P* = 1.8*10^–15^) and it is 0.10 (*P* = 5.10*10^–5^) for non-housekeeping genes. The overall Spearman correlation is -0.08 (*P* = 5.46*10^–9^). (**H**) Average poly(A) tail length per chromosome and for all autosomes plotted against average RNA half-life in S2 cells. All error bars represent the 95% confidence interval of the mean.

We and others have previously shown that, in mammalian cells, RNA stability correlates with basic characteristics of individual RNAs, such as transcript levels, RNA length and poly(A) tail length (18,45,47,48). mRNA stability has also been shown to correlate with RNA levels in *S. pombe* and *S. cerevisiae* but not in *E. coli* (45). We therefore asked whether these correlations are conserved during evolution and observable in *D. melanogaster* too. To assess the relationship between transcript levels and RNA stability, the genes were divided into 5 bins of equal numbers, based on their increasing transcript levels. We then calculated the average RNA half-life for the autosomes and the X-chromosome separately and for each cell line (Figure 2A). In contrast to what we previously observed in mammalian cells (18), for most bins there is no significant difference in average RNA half-life between autosomal transcripts and X-chromosomal transcripts. We observe a general positive correlation between RNA half-life and gene expression, in both male (S2) cells and female (Kc167) cells. To detect any potential chromosome-specific difference in RNA stability, we also plotted the chromosome-wise average for RNA half-life and RNA levels for males (S2 cells) (Figure 2B) and females (Kc167 cells) (Figure 2C). We observed less variation in average steady state transcript levels between chromosomes in females (Kc167 cells) compared to males (S2 cells) and in our experimental conditions the female transcript levels are overall slightly lower. The chromosome-wise averages also follow the positive correlation trend observed at the gene level, i.e. the average chromosomal transcript levels correlate positively with the average RNA half-life.

Next, to assess the relationship between gene length and RNA stability, we divided the genes into 5 bins of increasing gene length and with an equal number of genes per bin. We further classified the genes as housekeeping and non-housekeeping. We then calculated the average RNA half-life for each bin (Figure 2D). We found that RNA half-life correlates negatively with gene length in both males (S2 cells) and females (Kc167 cells) which is consistent with results obtained in mammals and *E. coli* but not in *S. cerevisiae* (18,48). To detect any potential chromosome specificity, we plotted the X-chromosomal and autosomal averages of RNA half-life and RNA length for male (S2) cells and female (Kc167) cells (Figure 2E and F). As expected, we observe that X-linked genes have on average longer RNAs but in contrast to the main autosome arms, this does not equate with significantly shorter half-lives even though the chromosome-wise Spearman correlations are within similar ranges. For the individual autosome arms, the chromosome-wise averages also follow the negative correlation trend observed at the gene level.

Finally, to assess the relationship between poly(A) tail length and RNA stability we divided the genes into 5 bins of increasing poly(A) tail length and with an equal number of genes per bin for males (S2 cells) (poly(A) tail length data for Kc167 cells were not available). We further classified the genes as housekeeping and non-housekeeping (Figure 2G). The results show that mRNA half-life correlates negatively with poly(A) tail length in housekeeping genes while the correlation is positive for non-housekeeping genes (Figure 2G). This contrasts with the positive correlation between poly(A) tail length and mRNA stability reported in human cells (49). The correlation for individual autosome arms between average poly(A) tail length and average RNA half-life is positive (Figure 2H).

We conclude that RNA stability correlates positively with steady state transcript levels and negatively with RNA length. Intriguingly, RNA stability correlates negatively and positively with poly(A) tail length for housekeeping and non-housekeeping genes, respectively.

### Knock-down of the MSL complex results in perturbations of both RNA levels and RNA stabilities

**Figure 3.**
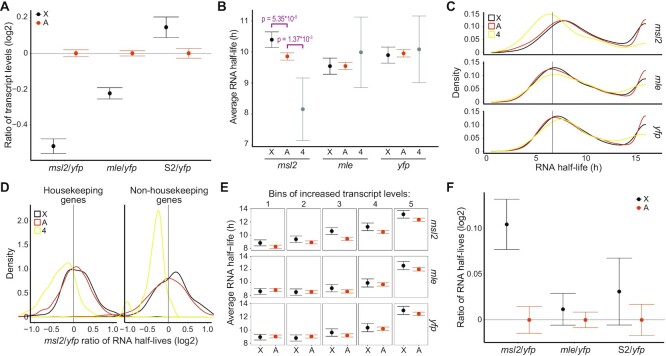
*msl2* knockdown leads to increased RNA stability of X-chromosomal transcripts and decreased RNA stability of fourth chromosomal transcripts. (**A**) Log2 ratios of transcript levels for the X-chromosome (black) and the autosomes (orange) with *yfp* RNAi as a reference. (**B**) Average RNA half-life for the X chromosome ‘X’, the main autosome arms ‘A’, and the fourth chromosome ‘4’, respectively, with *msl2*, *mle* and *yfp* RNAi. The *P*-values correspond to the Mann–Whitney *U*-test. (**C**) Density plots of RNA half-lives for all three RNAi experiments and for the X-chromosome (black), the main autosome arms (orange) and the fourth chromosome (yellow). The peak at 16 h represents all the RNAs that have a half-life equal to or above 16 h. (**D**) Density plots showing ratios of half-lives between the *msl2* RNAi sample and the *yfp* RNAi sample on log2 scale, separated between housekeeping genes (left) and non-housekeeping genes (right) for the X-chromosome (black), the autosomes (orange) and the fourth chromosome (yellow). (**E**) Average RNA half-life for the X-chromosome (black) and the autosomes (orange), in all three RNAi samples, for bins with equal numbers of genes with increasing transcript levels (GeTMM). (**F**) Log2 ratios of RNA half-lives for the X-chromosome (black) and the autosomes (orange) with *yfp* RNAi as a reference. S2 represents S2 cells without any RNAi treatment. All error bars indicate the 95% confidence interval of the mean.

We have previously shown that differential RNA stability between chromosomes contributes to dosage compensation in mammals (18). We therefore decided to investigate further the role, if any, of the MSL complex in the regulation of RNA stability in *D. melanogaster* even though we did not find any statistically significant difference in RNA half-life between the X-chromosome and the autosomes. To this end we knocked down two components of the MSL complex using RNAi: *msl2* encoding the core component MSL2 which is required for the complex to form, and *mle* encoding the RNA helicase MLE whose absence decreases proper spreading of the complex (21). The RNAi efficiency was confirmed by western blot and RNA-seq (Supplementary Figure S1 and Supplementary Table S2). As expected, both knock-downs cause a significant relative decrease in steady state RNA levels from the male X-chromosome, in line with impaired dosage compensation, and the effect is stronger with *msl2* RNAi as compared to *mle* RNAi (Figure 3A).

To test whether RNA stability is affected by an impaired MSL complex, we calculated the average RNA half-lives for the X-chromosome, the fourth chromosome, and the autosomes after RNAi knockdown (Figure 3B). Surprisingly, depletion of MSL2 significantly increases the average RNA half-life of the X-chromosome transcripts and causes a striking, significant decrease in average RNA half-life of the fourth chromosome transcripts compared to the autosomal transcripts. The depletion of MLE (which causes a lesser decrease in X-chromosome transcript levels) does not result in any statistically significant difference in RNA half-life between the X-chromosome and the autosomes, nor between the fourth chromosome and the autosomes (Figure 3B). The density plots of half-lives for the X-chromosome, the autosomes and the fourth chromosome show clear destabilization of chromosome 4 transcripts with *msl2* RNAi, while the distributions of half-lives for both the X-chromosome and the autosomes have shifted toward more stable half-lives, the X-chromosome's shift being larger (Figure 3C). Next, we separated the housekeeping genes from the non-housekeeping genes and plotted the distribution of the log2 differences in RNA half-life between the *msl2* RNAi sample and the *yfp* RNAi sample to test whether one of the two groups was causing the shift (Figure 3D). The results suggest that the stabilization of the X-chromosome transcripts is driven mainly by non-housekeeping genes. We then checked whether the stabilization of X-chromosome transcripts was over-represented among genes having similar steady state transcript levels. We split all genes into 5 bins of equal numbers of genes and increasing transcript levels and plotted the average RNA half-life for the X-chromosome and the autosomes (Figure 3E). Similar to the results obtained from untreated S2 cells (Figure 2A), the RNA stability correlates with the transcript levels but the relative stabilization of transcripts of the X-chromosome is observed only in MSL2 depleted cells (Figure 3E).

Finally, we calculated the log2 ratios of half-lives of our samples, using *yfp* RNAi as a reference (Figure 3F). We note that the RNAi treatment itself causes a slight relative increase in the half-life of the X-chromosome transcripts. However, *msl2* RNAi results in a larger and statistically significant increase in the X-chromosome half-lives while *mle* RNAi does not reveal any convincing difference.

We conclude that impairing the function of the MSL complex via *msl2* knock-down leads to a relative increase in the chromosomal average RNA half-life of the X-chromosome’s transcripts and a relative decrease in the chromosomal average RNA half-life of the fourth chromosome’s transcripts specifically. Additionally, the non-housekeeping genes of the X-chromosome seem to drive the shift in RNA stability.

### RNA stability counteracts induced transcriptional disturbances and enhances adapted differential transcription both in *Drosophila* and human cells

The shift in RNA stability observed with *msl2* RNAi (Figure 3B) could be explained by two different scenarios. First, higher stability of RNAs from the X-chromosome compared to autosomes may have had a role in dosage compensation prior to the existence of the chromosome-specific targeting of a fully functional MSL complex and what we observe are the evolutionary remains of such an ancient function. If so, the differential RNA stabilities should be specific to the X-chromosome. Alternatively, RNA stability may counteract induced changes in transcription output regardless of chromosome and the observed larger effect on the X-chromosome in *msl2* RNAi compared to autosomes is due to the fact that knock-down of MSL2 mainly down regulates transcription from the X-chromosome.

**Figure 4.**
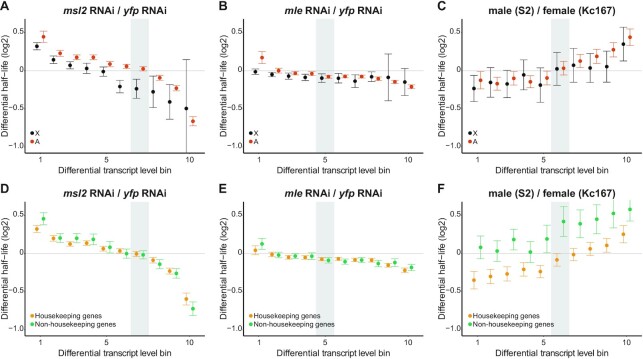
RNA stability counteracts induced transcriptional disturbances and contributes to adapted differential transcript levels. (**A**) Average differential RNA half-life (log2 scale) for bins of increasing differential transcript level and equal numbers of genes, for the X-chromosome (black) and the autosomes (orange), for comparison between the *msl2* RNAi sample over the *yfp* RNAi sample. The Spearman correlation coefficients are -0.51 (*P* = 1.47*10^–57^) and -0.61 (*P* < very low) for the X-chromosome and the autosomes respectively. (**B**) Comparison between the *mle* RNAi sample and the *yfp* RNAi sample. The Spearman correlations coefficients are -0.15 (*P* = 1.85*10^–5^) and -0.31 (*P* = 5.56*10^–31^) for the X-chromosome and the autosomes respectively. (**C**) Comparison between the S2 cell (male) sample without RNAi and the Kc167 cell (female) sample without RNAi. The Spearman correlation coefficients are 0.15 (*P* = 3.35*10^–5^) and 0.20 (*P* = 5.56*10^–31^) for the X-chromosome and the autosomes respectively. (**D**) Average differential RNA half-life (log2 scale) for bins of increasing differential transcript level and equal number of genes, for housekeeping genes (yellow) and non-housekeeping genes (green), for comparison between the *msl2* RNAi sample and the *yfp* RNAi sample. The Spearman correlations coefficients are -0.56 (*P* < very low) and -0.63 (*P* < very low) for the housekeeping genes and non-housekeeping genes respectively. (**E**) Comparison between the *mle* RNAi sample and the *yfp* RNAi sample. The Spearman correlations coefficients are -0.26 (*P* = 3.8*10^–90^) and -0.31 (*P* = 1.66*10^–74^), for the housekeeping genes and non-housekeeping genes respectively. (**F**) Comparison between the S2 cell (male) sample without RNAi and the Kc167 cell (female) sample without RNAi. The Spearman correlation coefficients are 0.23 (*P* = 6.74*10^–61^) and 0.16 (*P* = 4.93*10^–16^) for the housekeeping genes and non-housekeeping genes respectively. In (A–F), the gray rectangle highlights the differential transcript level bin that includes the value zero (no differential transcript level). All error bars represent the 95% confidence interval of the mean.

To test these two different hypotheses, we took advantage of the fact that *msl2* RNAi leads to globally differential transcript levels, mainly affecting the X-chromosome but indirectly also the autosomes. We therefore compared differential RNA stabilities to differential transcript levels. We divided all genes into 10 bins of equal numbers of genes, based on increasing differential transcript levels, and calculated the average differential RNA half-life for the X-chromosome and the autosomes (Figure 4A–C), the housekeeping and non-housekeeping genes (Figure 4D–F), quartiles of increasing RNA length (Supplementary Figure S3A–C) and quartiles of increasing poly(A) tail length (Supplementary Figure S3D–F).

Comparing *msl2* RNAi to *yfp* RNAi, we observe a consequent and statistically significant negative correlation between differential RNA stabilities and differential RNA levels, for both the X-chromosome and the autosomes (Figure 4A) as well as for housekeeping and non-housekeeping genes (Figure 4D). The negative correlation holds true when genes are separated into quartiles of increasing RNA length or poly(A) tail length (Supplementary Figure S3A and S3D, respectively). The results show that, upon induced transcriptional differences using *msl2* RNAi, RNA under-transcription is counteracted by RNA stabilization, and reciprocally, RNA over-transcription is counteracted by RNA destabilization. We observed similar correlations for *mle* RNAi, although, as expected the amplitude was reduced (Figure 4B and E; Supplementary Figure S3B and E). Next, to check if similar correlations could be found in mammalian cells we used a BRIC-seq data set with depletion of UPF1 (Upstream frameshift 1) (50). UPF1 is an ATP-dependent RNA helicase and a core factor of nonsense-mediated mRNA decay (NMD) (51,52). It has previously been shown that *UPF1* RNAi causes global changes in both RNA stabilities and transcription (18,36,53,54) and we therefore asked whether the RNAi-induced global differences in RNA stability and RNA levels correlate. In line with the results obtained in *Drosophila*, we observed a general negative correlation between differential RNA levels and differential RNA stability upon knockdown of UPF1 in HeLa cells (Supplementary Figure S4A) that holds true for housekeeping and non-housekeeping genes (Supplementary Figure S4B).

Our results show that differential RNA stability counteracts induced disturbances in transcription output both in *Drosophila* S2 cells and mammalian HeLa cells. Conversely, it has been shown in yeast that strains deficient for mRNA degradation factors compensate the dysfunctional decay by altering their mRNA synthesis rates (55).

We next asked whether and how RNA stability differs between male and female cells for genes with assumed adapted differential expression. To do this, we plotted the differential transcript levels between males (S2 cells) and females (Kc167 cells) against their differential RNA stability values (Figure 4C and F; Supplementary Figure S3C and F). Intriguingly, we observe a positive correlation in this case, which means that genes with lower RNA levels in male cells compared to female cells also have less stable RNAs, while genes whose RNAs are more abundant in male cells compared to female cells have more stable RNAs, for both the X-chromosome and the autosomes (Figure 4C) and for the housekeeping and non-housekeeping genes (Figure 4F). To test if the observed relationship between RNA stability and adapted RNA levels holds true also in mammals we analyzed available data from 7 lymphoblastoid cell lines (37). We plotted differential RNA stability for 10 bins of increasing differential expression, for the X-chromosome and the autosomes, pairwise using all cell lines (3 females and 4 males) (Supplementary Figure S4 C-W). We observe positive correlations of varying degrees for both the X-chromosome and the autosomes in all pairwise comparisons. Notably, the correlations are observed both for male-female comparisons as well as comparisons of two different lines of the same sex (Supplementary Table S5).

By correlating differential half-life with differential RNA levels in different conditions, we draw two conclusions. First, the regulation of RNA stability counteracts induced transcriptional disturbances in both autosomes and sex chromosomes, in both flies and mammals. Second, it contributes to the adapted differential transcript levels between S2 cells and Kc167 cells but also between any two independent human lymphoblastoid cell lines.

### Ribosome density varies across chromosomes and gene functions

We have previously shown that, in mammals, RNA stability as well as ribosome density are significantly higher for the X-chromosome compared to autosomes and that these two factors contribute to dosage compensation (18). As RNA stability doesn′t seem to contribute to dosage compensation in *D. melanogaster*, we asked how ribosome density relates to RNA stability. To address this question, we analysed paired ribo-seq and RNA-seq data from (30) and calculated ribosome densities for all expressed genes.

**Figure 5.**
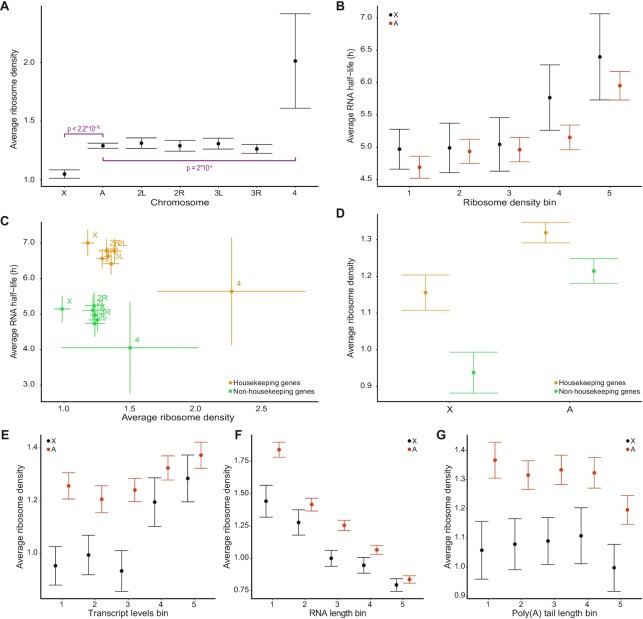
Chromosome specific and gene function specific regulation of translation and correlation with RNA half-life. (**A**) Average ribosome density per chromosome in male (S2) cells. The *P*-values indicated were calculated for pairwise comparisons using a Wilcoxon rank sum test with continuity correction. (**B**) Average RNA half-life for bins of increasing ribosome density and equal number of genes, for the X-chromosome and the autosomes. The Spearman correlation is 0.16 (*P* = 1.24*10^–33^) for the X-chromosome and 0.13 (*P* = 4.5*10^–70^) for the autosomes. (**C**) Average ribosome density per chromosome plotted against average RNA half-life, split between housekeeping genes and non-housekeeping genes, for S2 cells. The Spearman correlations for gene groups are 0.16 (*P* = 2.06*10^–4^) for the housekeeping genes of the X-chromosome; 0.13 (*P* = 1.51*10^–11^) for the housekeeping genes of the autosomes; 0.05 (*P* = 0.33) for the non-housekeeping genes of the X-chromosome; 0.06 (*P* = 0.022) for the non-housekeeping genes of the autosomes. (**D**) Average ribosome density for the X-chromosome and the autosomes, separated between housekeeping genes (yellow) and non-housekeeping genes (green). (**E**) Average ribosome density for bins of increasing transcript levels (TPM) and equal numbers of genes, for the X-chromosome (black) and the autosomes (orange). The Spearman correlations between ribosome density and RNA levels are 0.36 (*P* = 1.24*10^–33^) and 0.24 (*P* = 4.50*10^–70^) for the X-chromosome and the autosomes respectively. (**F**) Average ribosome density for bins of increasing RNA length and equal numbers of genes, for the X-chromosome (black) and the autosomes (orange). The Spearman correlations are -0.40 (*P* = 3.27*10^–42^) and -0.47 (*P* = 1.04*10^–286^) for the X-chromosome and for autosomes respectively. (**G**) Average ribosome density for bins of increasing poly(A) tail length and equal numbers of genes, for the X-chromosome (black) and the autosomes (orange). All error bars represent the 95% confidence interval of the mean.

We found that the average ribosome density is significantly lower for the X-chromosome compared to the autosomes (Figure 5A). This result contrasts with our previous findings in mammalian cells (18) but is in agreement with a *Drosophila* study reporting that X-linked transcripts have approximately 20% lower ribosome densities than autosomal transcripts, not only in S2 cells but also in early embryos, eggs and mature oocytes (56). Here, we analyzed data from S2 cells only, using matching ribo-seq and RNA-seq datasets originating from the same laboratory and cell stock, and found an average of 23% lower ribosome density for the X-chromosome compared to the autosomes. In addition, we observed that the average ribosome density is significantly higher for the fourth chromosome compared to the autosomes (Figure 5A). More specifically, there are on average 1.05, 1.29 and 2.01 ribosomes per kilobase of transcript for genes encoded on the X-chromosome, the autosomes, and the fourth chromosome respectively (Supplementary Figure S5).

Next, to characterize the relationship between ribosome density and RNA stability, we divided all genes where there were data for both RNA stability and ribosome density into 5 bins with equal numbers of genes and increasing ribosome density. We further divided the genes into autosomes and X-chromosome and plotted the groups’ average RNA half-lives (Figure 5B). We observe a positive correlation between half-life and ribosome density for both the X-chromosome and the autosomes. Plotting chromosome-wise average RNA half-lives against chromosome-wise average ribosome densities grouped by chromosome and housekeeping status shows a proportionally higher RNA half-life and higher ribosome density for the housekeeping genes on each chromosome compared to the non-housekeeping genes (Figure 5C and D).

To delineate potential correlations between ribosome density and transcript levels, we divided the genes into 5 bins based on increasing transcript levels (TPM calculated using the RNA-seq data coupled with the ribo-seq data) and calculated the average ribosome density per bin for the autosomes and the X-chromosome (Figure 5E). We note a positive correlation for both the X-chromosome and the autosomes, and the average ribosome density is significantly different between the X-chromosome and the autosomes for genes with low to medium transcript levels (bins 1–3) (Figure 5E).

Given that mRNAs from the X-chromosome are on average longer compared to mRNAs from the autosomes and that generally, the ribosome density decreases as the RNA length increases, we asked whether this could explain the lower ribosome density observed for the X-chromosome. We therefore compared ribosome density averages for bins with identical transcript length ranges (Figure 5F). Our results indicate that the different average mRNA lengths between the X-chromosome and the autosomes do not cause the difference in ribosome density because the ribosome density of the X-chromosome transcripts is still lower compared to autosomes within the same mRNA length bin. Additionally, we find that the correlation between ribosome density and mRNA length is statistically significant for both the X-chromosome and the autosomes.

Finally, it has been shown that changes in poly(A) tail length participate in translational regulation and that poly(A) shortening acts as a timer for RNA decay (57). We therefore checked whether the lengths of poly(A) tails correlate with ribosome densities. We divided the genes into 5 bins based on increasing poly(A) tail length and calculated the average ribosome density per bin for both the X-chromosome and the autosomes (Figure 5G). We find that the average ribosome density for the autosomes is significantly different from the average ribosome density for the X-chromosome for all poly(A) tail length bins (Wilcoxon rank sum test, all *P* < 0.05) and that there is no obvious correlation between the two variables (Figure 5G).

We conclude that ribosome density is greater on transcripts from housekeeping genes compared to transcripts from non-housekeeping genes and it is consistently lower on transcripts of the X-chromosome compared to transcripts of the autosomes. This decreased ribosome density on transcripts of the X-chromosome is not explained by X-specific gene characteristics. Intriguingly, we observe a significantly higher average ribosome density for the fourth chromosome.

## DISCUSSION

Three main conclusions can be drawn from our analysis. First, the modulation of RNA stability counteracts widespread, acute disturbances in transcription output by increasing half-life upon down-regulation of transcription and vice versa both in flies and mammalian cell lines. Second, differential gene expression between two distinct cell lines is partly mediated via modulation of RNA stability, both in flies and mammals. Third, contrary to what has been reported in mammals, there is no evidence for a role of RNA stability regulation in dosage compensation of the sex chromosomes in flies.

### Physiological genome-wide RNA stability in *Drosophila melanogaster*

The BRIC-seq method (28) is superior to transcriptional shut-off methods using drugs such as Actinomycin-D since it measures the RNA stability in cells under normal physiological conditions (58). We observed that transcripts from the female cell line (Kc167) are consistently more stable than those from the male cell line (S2). This difference may result from a cell-type difference and may not be dependent on the sex. Moreover, it has been reported that no two cell lines have similar expression patterns, and they reflect, with some attenuation, the expression patterns of the individual cells from which they originate, combined with the consequences of chromosomal rearrangements they underwent to reach immortality (59,60). In addition, it is likely that the BrU labeling was more stressful to the Kc167 cells than the S2 cells; indeed, we observed cell toxicity at lower BrU concentrations in Kc167 compared to S2 cells and this could slightly alter global RNA stabilities. In support of this hypothesis, it has recently been shown that stress conditions leading to global transcription attenuation such as UV exposure can provoke general stabilization of cellular mRNAs in mammals (61). In our case, the incorporation of BrU into transcripts could potentially affect transcription dynamics, and thereafter RNA stability.

We show that RNA stability is linked to gene function, which is in line with previous observations in mammals and yeast, *i.e*. housekeeping genes have greater RNA stability compared to genes having functions in e.g. transcription regulation or apoptosis (28,43,46). Differential RNA stability of individual gene transcripts is beneficial for the cell as it allows rapid changes in key RNA levels in response to changing conditions while saving the energy required to renew transcripts that are meant to be expressed continuously at stable levels (2,43,62). Our results expand the relationship between RNA stability and gene function to flies and suggest that it is conserved across species.

Finally, contrary to the results obtained in mammals (18), we did not find any convincing difference between the stability of the X-chromosome and autosomal transcripts, neither in male cells (S2) nor in female cells (Kc167), which indicates that the role of RNA stability in dosage compensation in *D. melanogaster* is at most minimal.

### Significantly higher RNA stability is observed for highly expressed genes, genes coding for shorter mRNAs and housekeeping genes with shorter poly(A) tail length

The correlation of RNA stability with transcript levels was expected, partly because RNA stability contributes to measured transcript levels. At equal transcription rates, a longer RNA half-life leads to higher RNA levels. Furthermore, RNAs that are constantly required at high levels are likely selected to be more stable, saving the energetic costs of transcribing and degrading transcripts.

We find that transcripts from long genes are in general less stable than those from short genes in *D. melanogaster*. It has been hypothesized that long genes are more likely to undergo mechanical damage or random endonucleolytic attacks than short genes simply due to their length (48). Additionally, long RNAs are statistically more likely to harbor transcription errors that would target them for decay. We found a slight but significant general negative correlation between RNA stability and poly(A) tail length in S2 cells, consistent with the findings in HeLa cells and 3T3 cells from (31). This negative correlation contradicts the prevailing idea that the longer a poly(A) tail the more stable the transcript (57). However, this view is being refined with the development of methods that measure poly(A) tail length genome-wide. It has been reported that mRNAs coding for ribosomal proteins or other housekeeping genes are enriched in shorter poly(A) tail lengths in humans, fly, yeast, zebrafish and plants (31,63). Interestingly, when we separated genes into housekeeping and non-housekeeping, it appeared that the two groups had an opposite, stronger correlation between RNA half-life and poly(A) tail length. Taken together, this suggests that the poly(A) tail length of housekeeping gene transcripts is regulated specifically. This is supported by (64), who discovered a sequence-encoded enhancer–core-promoter specificity that separates the regulatory programs of developmental genes from those of housekeeping genes in *Drosophila*. It is plausible that these transcriptional programs include factors influencing the length and modifications of the poly(A) tail of the transcript that they activate, and thus carry information about the associated stability. Moreover, it has been proposed that short-tailed housekeeping genes could be locked in a closed loop state that promotes translation and stabilizes the RNA, favoring constitutive expression (63).

### The modulation of RNA stability buffers RNA levels genome-wide upon induced changes in transcription output and participates to differential RNA expression between individuals

We describe two RNA stability mediated responses to alterations in gene expression. The first one buffers sudden perturbations in transcription by shifting RNA stability in the opposite direction of the transcriptional change and was observed both in insect and mammalian cells. Our finding is supported by a recent study in mammals that found that global alterations in transcriptional dynamics led cells to rapidly and specifically adjust the expression of their RNA degradation machinery in order to counteract the changes and buffer mRNA levels (61). Moreover, our observations are in line with previous observations of buffering in *D. melanogaster* (4–6). The response of RNA stability to transcript level change appears more pronounced for non-housekeeping genes compared to housekeeping genes. Interestingly, we have previously shown using microarray analyses in flies that in monosomic regions, non-housekeeping genes are more strongly buffered than housekeeping genes, i.e. their RNA levels are closer to the wild-type levels (5,65). This parallel suggests that upon impairment of the MSL complex, the same, general, non-MSL-complex-dependent buffering mechanism still operates (or takes over), prioritising stabilization of the RNAs of non-housekeeping genes, that are often short-lived in relation to their function. We speculate that changes in RNA stability constitute a general response that partially buffers acute disturbances in transcript levels such as those resulting from loss of a chromosome segment, an impaired dosage compensation mechanism or a new mutation that broadly alters RNA levels. There is evidence that mRNA decay is coupled to translation, which is coupled to mRNA export, maturation and transcription (66–68), and these biological links could constitute the foundations for a mechanism that modulates RNA stability in relation to changes of its transcription output. The increase in RNA stability upon loss of MSL2, buffers the RNA levels and may explain why, experimentally, a knockdown of the MSL complex does not result in a two-fold decrease in transcript levels (6,38–41,69). The increased RNA stability is not sufficient to rescue lost MSL complex function but might have been enough to transiently alleviate the negative phenotypes associated with the progressive degeneration of the Y-chromosome. This way it could have allowed the evolution of the more effective and specific MSL complex dependent mechanism.

The second response supports and strengthens the inter-individual differential gene expression. We correlated differential RNA stability with differential RNA levels of two embryo derived insect cell lines S2 (male) and Kc167 (female) cells and of seven human lymphoblastoid cell lines of mixed sexes. The RNA stability response shows no sex-chromosome bias and leads to greater stability in the case of genes exhibiting higher RNA levels in a specific cell line. This response (in contrast to buffering that minimizes the differences in transcript levels) will contribute to the differential transcript levels measured here. Finally, we speculate that RNA stability could also be used among other mechanisms to control adaptive expression during speciation.

The fourth chromosome is much smaller than the other chromosome arms and has fewer genes, thus it is challenging to detect significant differences compared to the other chromosomes. Still, we find that with *msl2* RNAi, the fourth chromosome has a much, and highly significantly, lower RNA stability compared to the autosomes. It is unclear as to why this happens, and we speculate that elements of the answer lie within one or several of its unique characteristics. The fourth chromosome is enriched in repetitive elements, it is replicated late, and in principle, the entire chromosome can be considered heterochromatic (70,71). Despite its heterochromatic nature, we and others have previously shown that average transcript levels from the fourth chromosome are comparable to, or even higher than, those of genes on other chromosomes (72,73). In addition, the fourth chromosome displays unusually high tolerance of dosage differences and mis-expression (5,73–76) and it has been shown that it was ancestrally an X-chromosome that has reverted to being an autosome (34,35,77).

### Gene expression from the X-chromosome and fourth chromosome is regulated at the translational level on a chromosome specific basis

We observe a lower ribosome density on transcripts from the X-chromosome in untreated male cells (S2) which confirms previous findings (56). They hypothesized that the reduced ribosome density is consistent with slower translation initiation and not faster translation elongation because X-linked transcripts have stronger mRNA structures near start codons and longer 5′UTRs, both features being known to slow translation initiation (56). The cited article also confirms previous findings that X-chromosome transcripts in *Drosophila* have a higher codon usage bias than those from other chromosomes (78). Codon optimality has been shown to facilitate translation elongation in yeast (79–81). Ribosome density is used here as a proxy for translational activity, but it is an imperfect measure because ribosome densities can be used to compare the translation efficiency of genes only if their translation elongation rates are equal (82). A recent study showed that optimal codons speed up translation elongation in a *Drosophila* cell-free system (83), and if this also occurs *in vivo*, it favours the faster translation elongation hypothesis for the X-chromosome transcripts. If translation elongation is indeed faster for transcripts from the *Drosophila* X-chromosome, this is in line with our observed lower ribosome density.

Additionally, we find a significantly higher ribosome density on the transcripts of the fourth chromosome. The fourth chromosome of *Drosophila* has a low codon usage bias and a high level of repetitive sequences and is highly heterochromatic (77). According to the findings of (83), a low codon usage bias slows down translation elongation and could thus explain the higher ribosome density we observe. In particular, a low ribosome density in untreated cells corresponds to increased RNA stability with *msl2* RNAi (X-chromosome) and a high ribosome density in untreated cells corresponds to decreased RNA stability with *msl2* RNAi (chromosome 4). It is plausible that in *msl2* RNAi cells, the reduced number of X-chromosome transcripts leads to increased translation initiation rates driven by feedback loop regulation processes, which in turn increases RNA stability. The opposite effect may apply for the fourth chromosome.

Ribosome densities on the X-chromosomal and the autosomal RNAs differ significantly for genes with low to medium transcript levels but not for genes with high transcript levels. This suggests that genes with low to medium transcript levels are more likely to be regulated at the translational level than genes with high transcript levels, because the latter are likely to be saturated at the translational level.

The chromosome-wise average RNA half-lives are not significantly different from each other; however, the ribosome densities are. Even though ribosome density correlates negatively with gene length, and the X-chromosome has, on average longer genes, for each gene length bin, the X-chromosome still has a lower ribosome density on its transcripts than the autosomes. This suggests that the chromosome specific regulation (dosage compensation) of gene expression is acting mainly at the transcriptional and translational levels rather than through the regulation of RNA stability in *Drosophila melanogaster*.

In summary, we postulate that the regulation of RNA stability constitutes a versatile tool for the cell, used to control gene expression in several ways. Firstly, it can be used rapidly to buffer shifts in transcription output, provoked by e.g. some mutation that results in broad alterations of transcription such as for general transcription factors or genes involved in dosage compensation, and thus minimize the effects of faulty transcription output. Second, the modulation of RNA stability has a supporting role to establish differential RNA levels between individuals or cell types genome-wide. Lastly, as shown in mammals by (2), it can be used to accelerate the operation of expression off-switches (developmental or in response to stress) by selectively increasing the rate of decay of unwanted RNAs.

## ACCESSION NUMBERS

The BRIC-seq and RNA-seq data reported in this paper have been deposited in the European Nucleotide Archive with accession number PRJEB15335.

## Supplementary Material

gkac208_Supplemental_Files
