# Supplementary material for: Modulation of RNA stability regulates gene expression in two opposite ways: through buffering of RNA levels upon global perturbations and by supporting adapted differential expression
Source: Nucleic Acids Res. 2022 Apr 7;50(8):4372–88. doi: 10.1093/nar/gkac208 (PMC9071389; doi:10.1093/nar/gkac208)
Supplement: gkac208_Supplemental_Files [file gkac208_supplemental_files.zip › Combined_Supplementary_figures_V4.pdf]

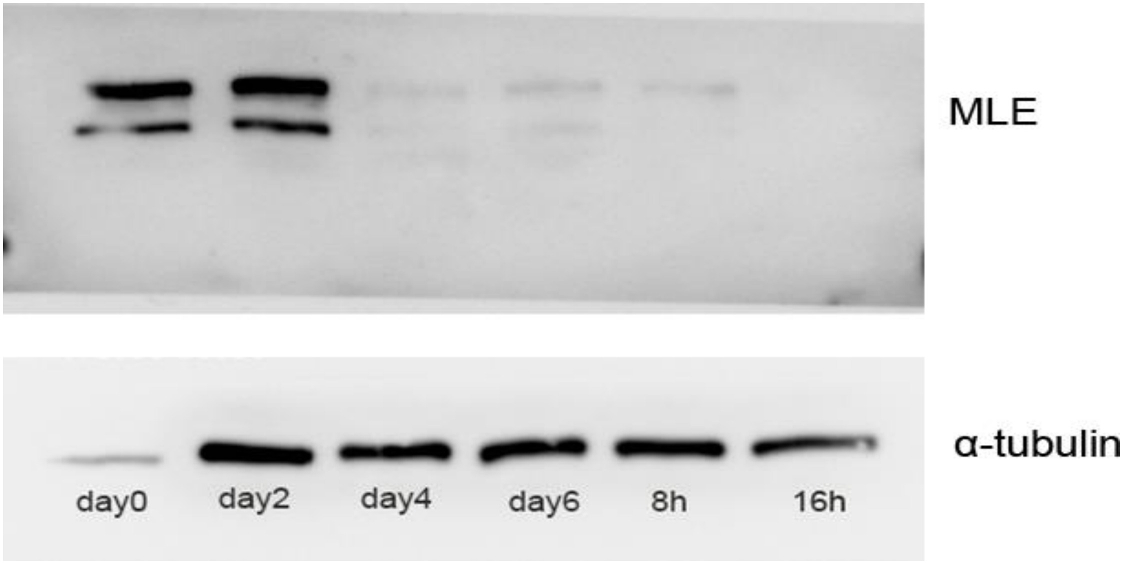

Supplementary figure S1. Confirmation of RNAi efficiency. Western blot for MLE during *mle* RNAi treatment. Day 0 corresponds to the amount of protein before the RNAi treatment, the other samples after the number of indicated days. At day 4, the RNAi treatment was renewed. Western blot with antibodies against α-tubulin serves as loading control.

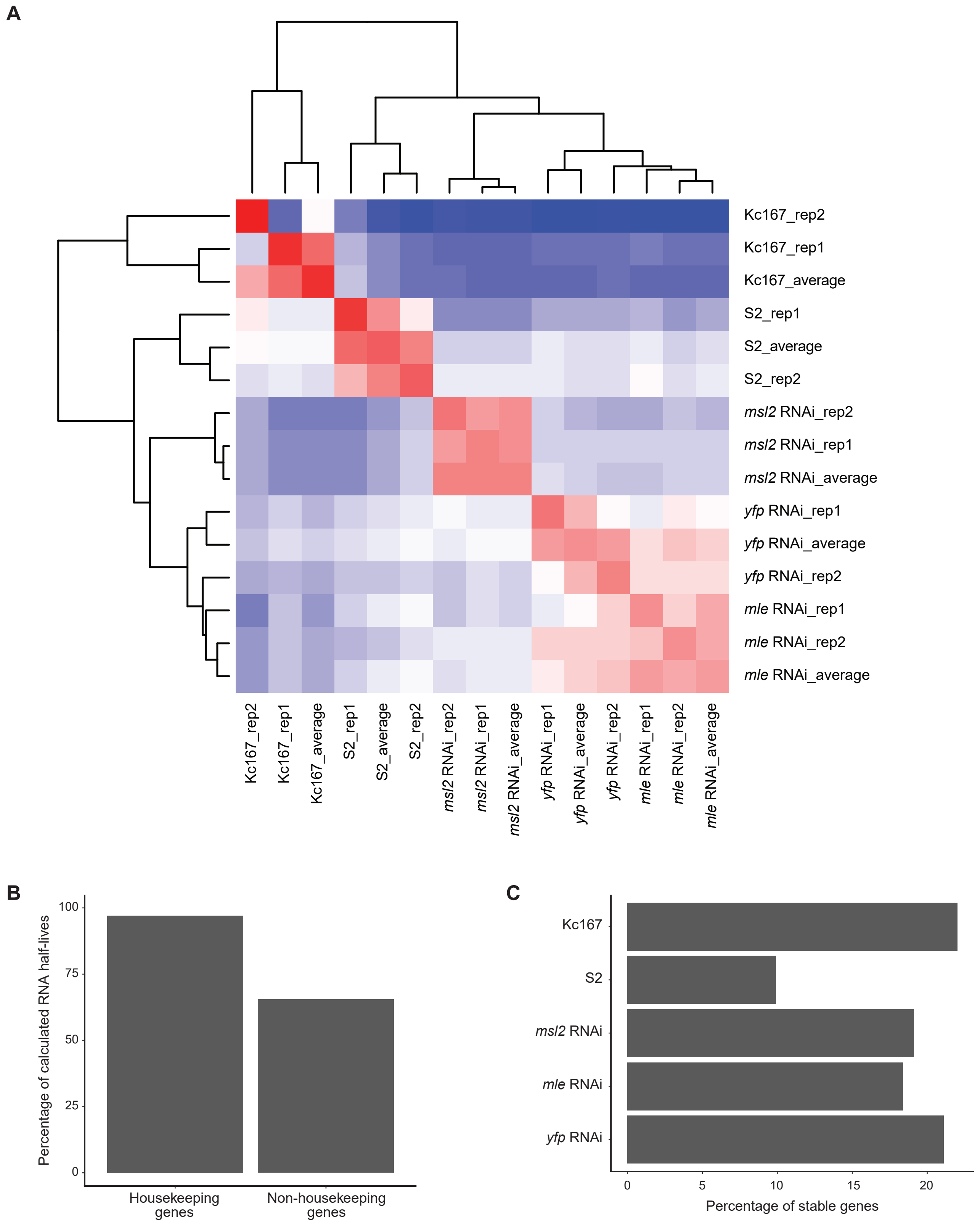

Supplementary figure S2. Dataset characterization (**A**) Correlation matrix for calculated half-lives. Red represents the highest correlations and blue represents the lowest correlations. (**B**) Percentage of housekeeping genes among expressed genes for which a half-life has been calculated in at least one sample. (**C**) Percentage of genes with a half-life of over 16 h (stable).

Supplementary Figure S2

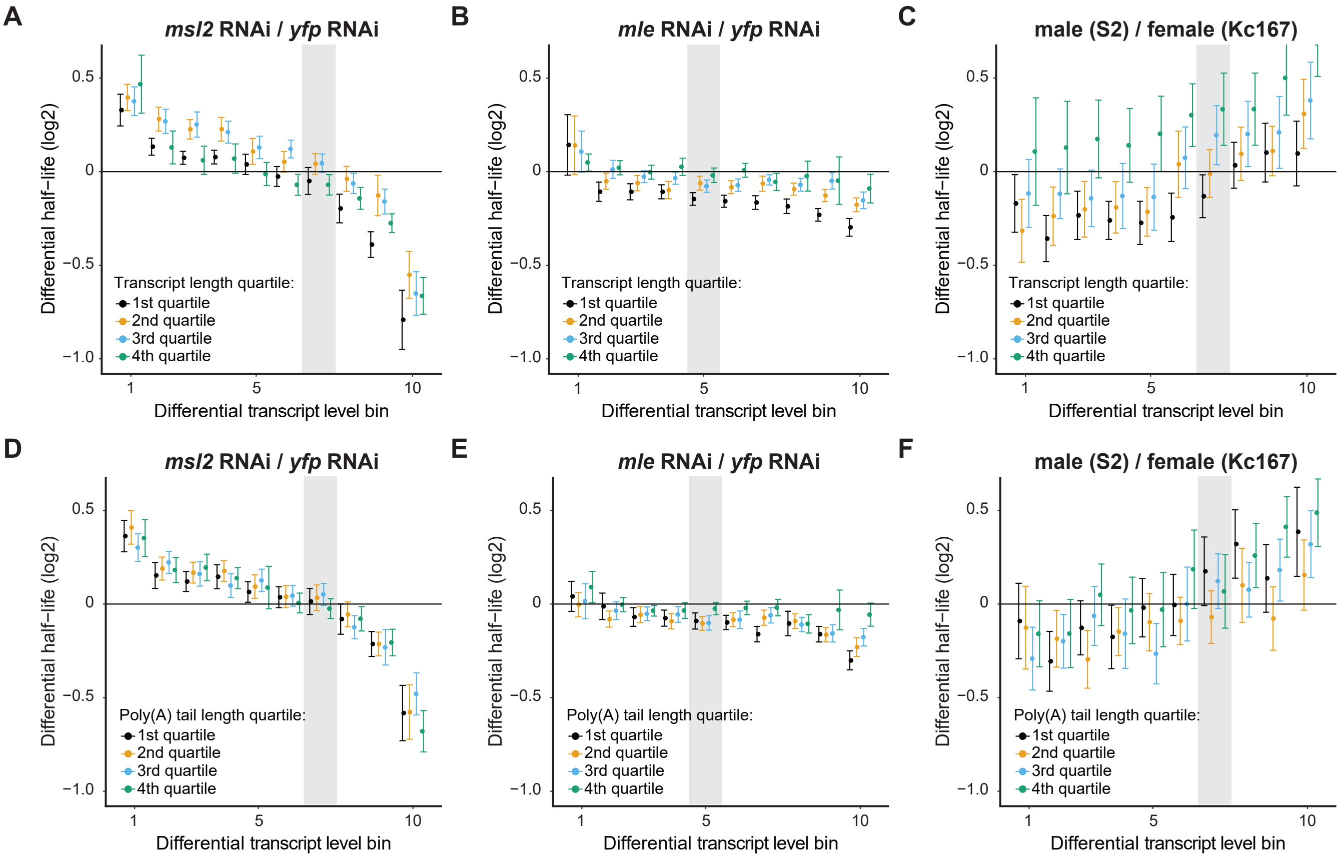

Supplementary figure S3. RNA stability counteracts induced transcriptional disturbances and contributes to adapted differential transcript levels in flies. (**A**) Average differential RNA half-life (log2 scale) for bins of increasing differential transcript level and equal numbers of genes, for the quartiles of increasing transcript length, for comparison between the *msl2* RNAi sample and the *yfp* RNAi sample. (**B**) Comparison between the *mle* RNAi sample over the *yfp* RNAi sample. (**C**) Comparison between the S2 cell (male) sample without RNAi and the Kc167 cell (female) sample without RNAi. (**D**) Average differential RNA half-life (log2 scale) for bins of increasing differential transcript level and equal numbers of genes for the quartiles of increasing poly(A) tail length, for comparison between the *msl2* RNAi sample and the *yfp* RNAi sample. (**E**) Comparison between the *mle* RNAi sample and the *yfp* RNAi sample. (**F**) Comparison between the S2 cell (male) sample without RNAi and the Kc167 cell (female) sample without RNAi. In graphs A-F, the grey rectangle highlights the differential transcript level bin that includes the value zero (no differential transcript level). All error bars represent the 95% confidence interval of the mean.

Supplementary Figure S3

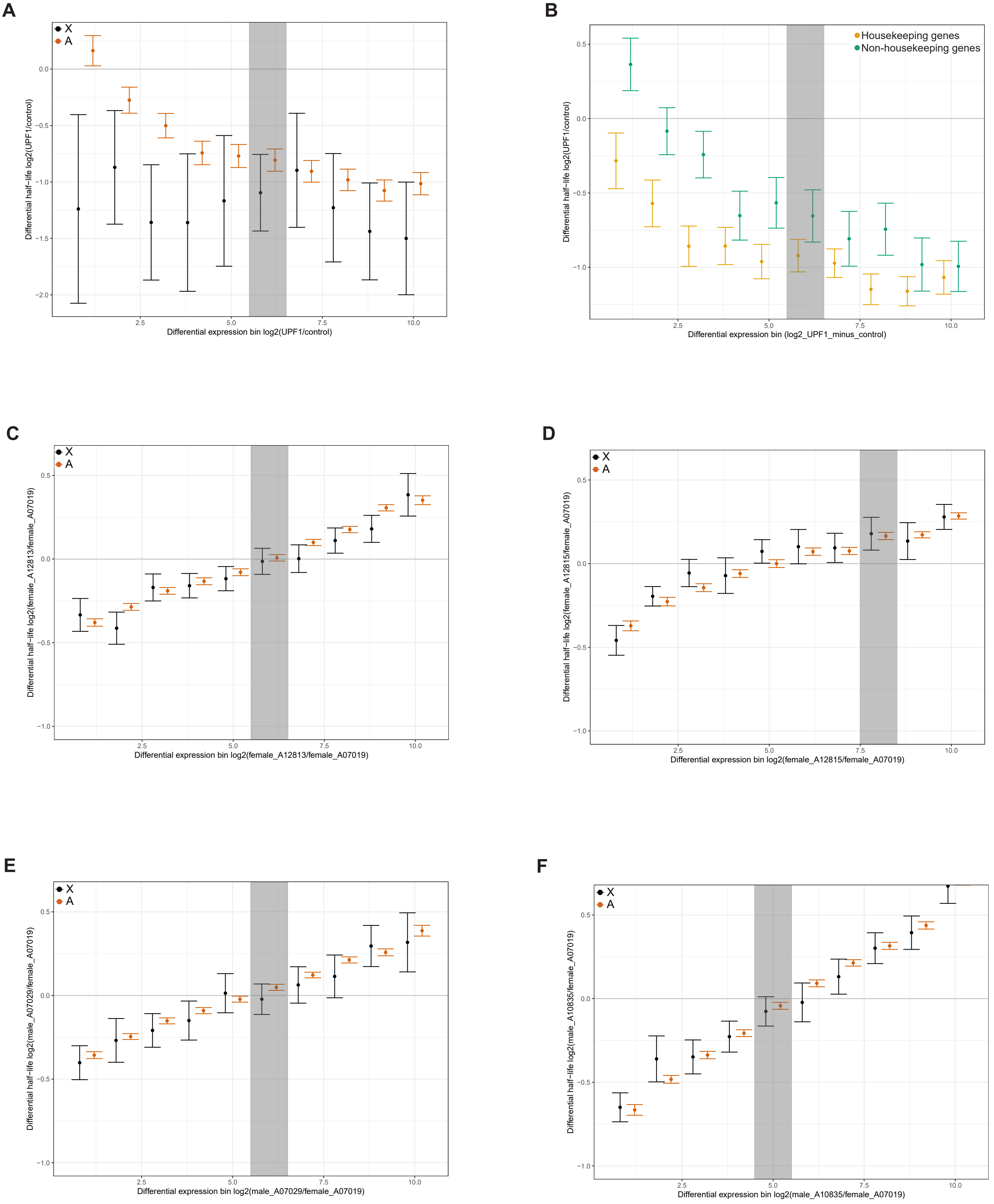

Supplementary Figure S4 (1/4)

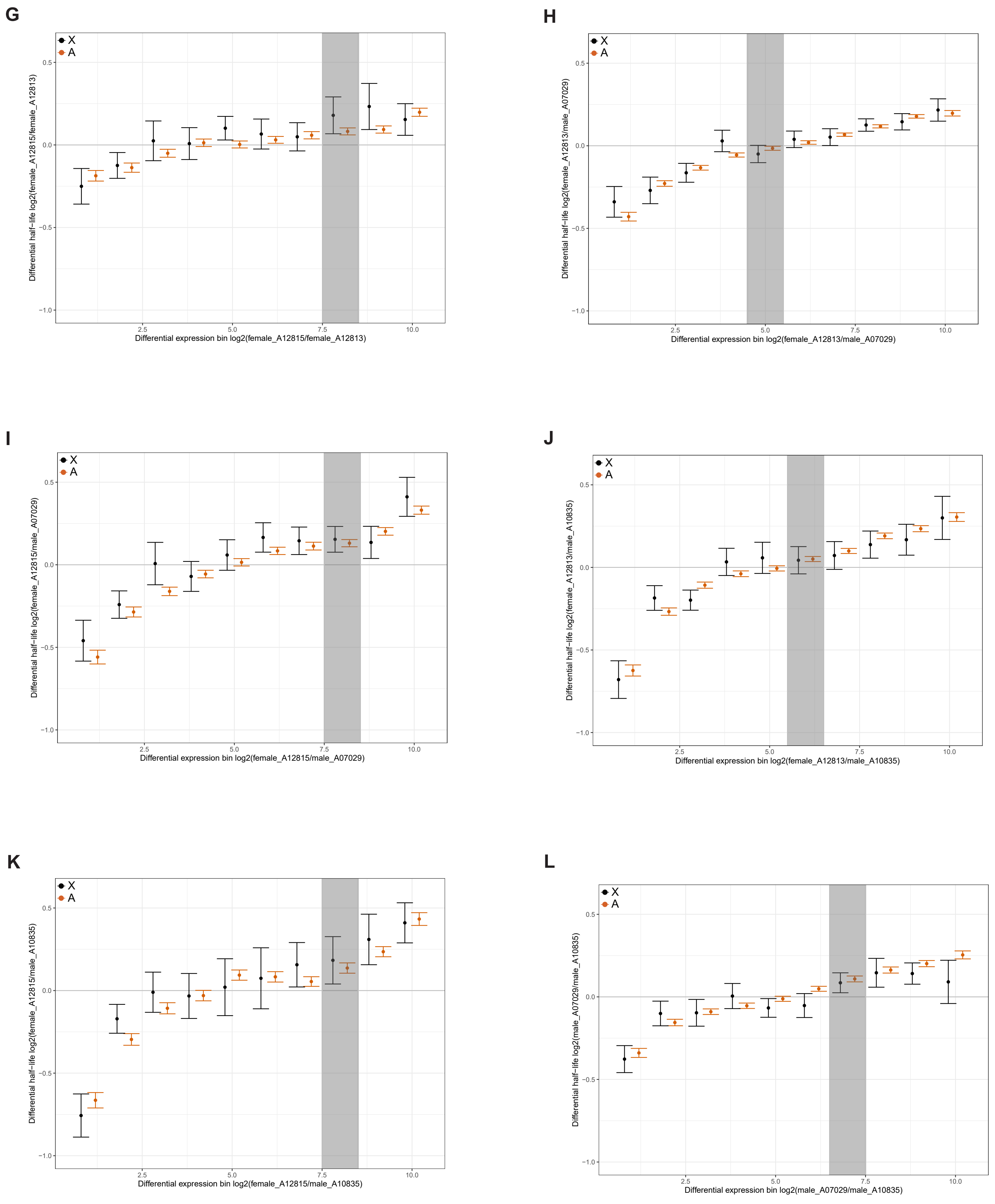

Supplementary Figure S4 (2/4)

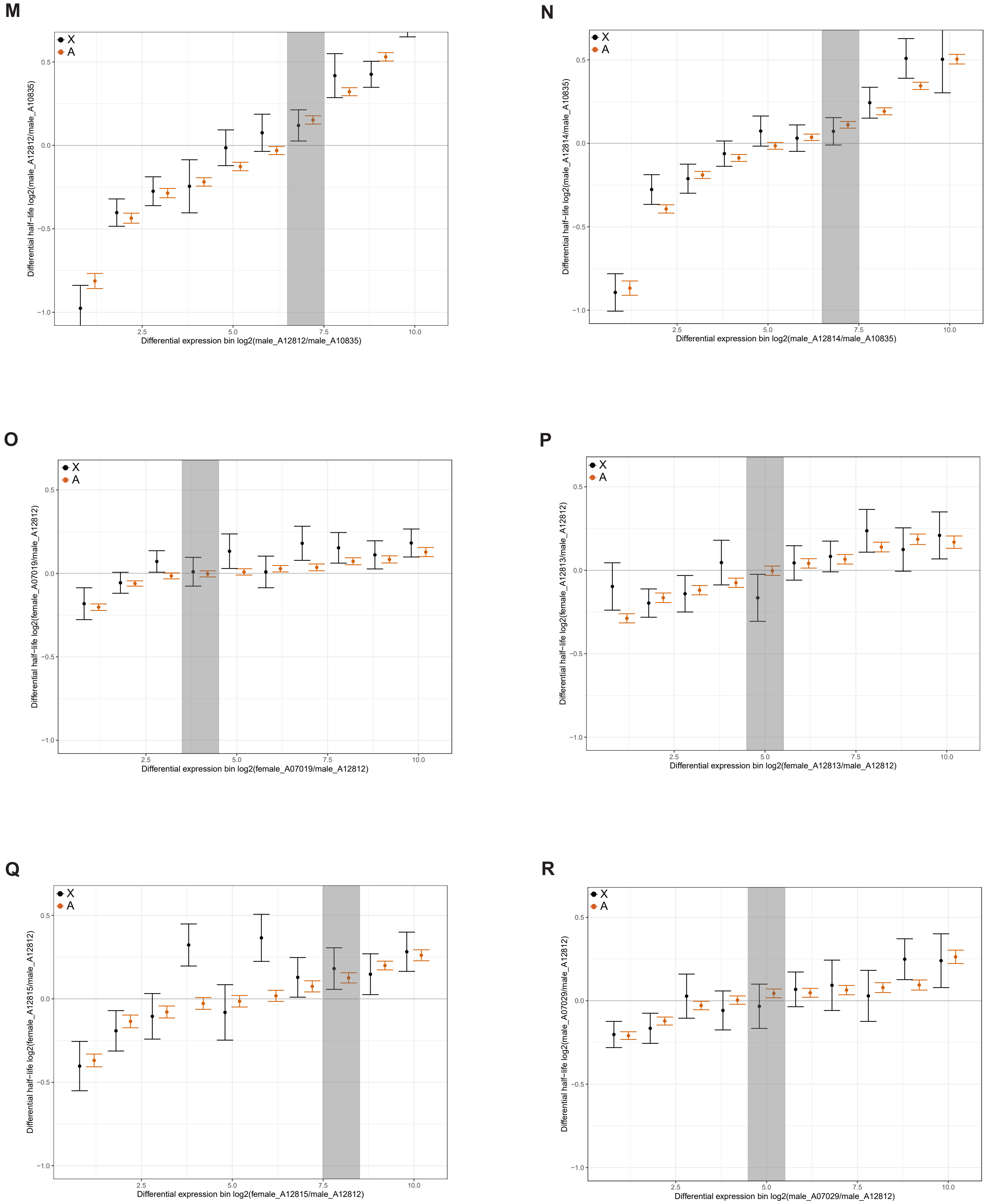

Supplementary Figure S4 (3/4)

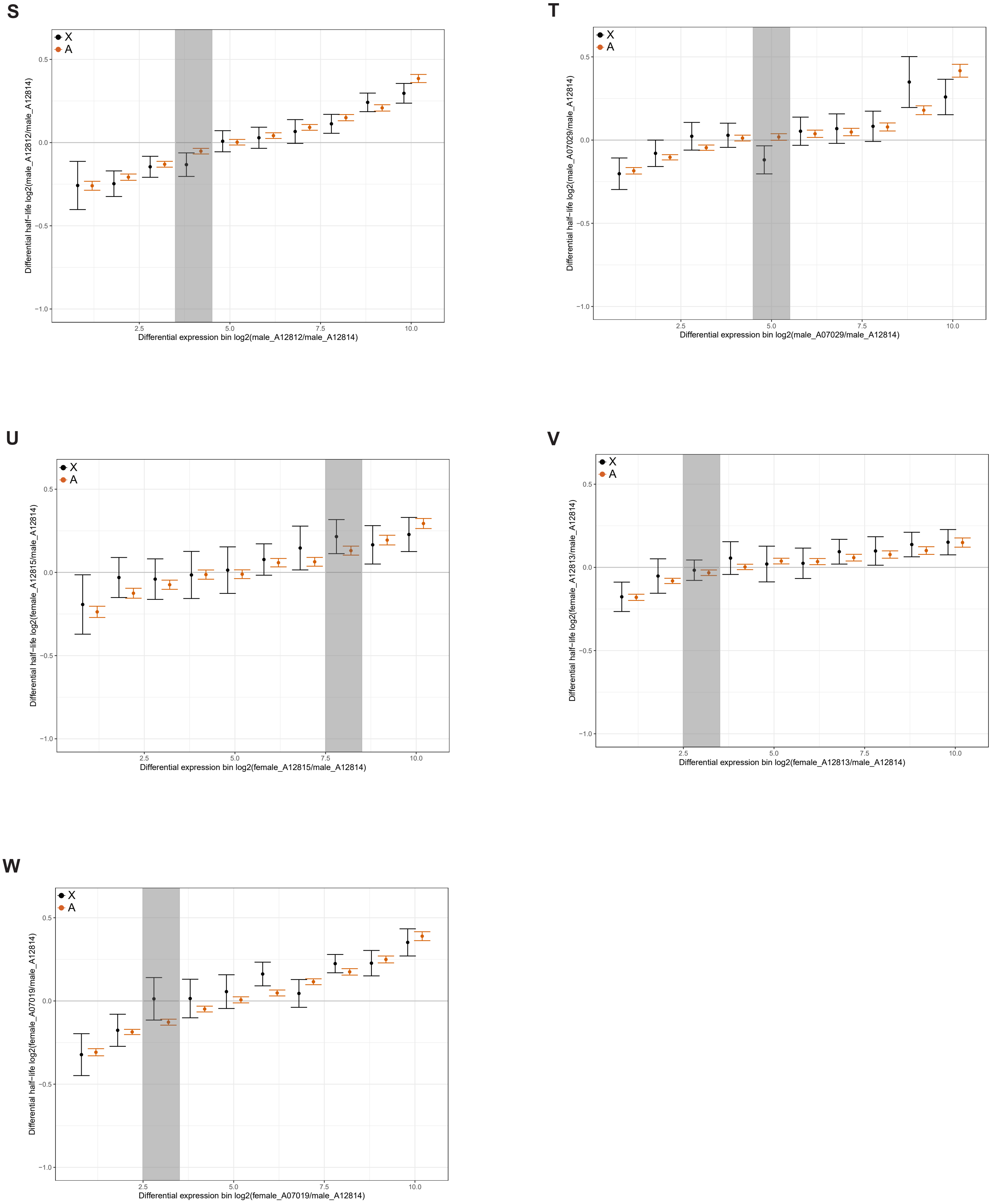

Supplementary figure S4. RNA stability counteracts induced transcriptional disturbances and contributes to adapted differential transcript levels in mammals. (**A**) Average differential RNA half-life (log2 scale) for bins of increasing differential transcript level and equal numbers of genes, for the X-chromosome (black) and the autosomes (orange), for comparison between a UPF1 knock-down and control in HeLa cells. The Spearman correlation coefficients are -0.067 ($p = 0.22$) and -0.20 ($p = 5.41*10^{-98}$) for the X-chromosome and the autosomes respectively. (**B**) Same as (A) but the genes are separated between housekeeping genes (yellow) and non-house-keeping genes (green). The Spearman correlations coefficients are -0.15 ($p = 5.80*10^{-55}$) and -0.21 ($p = 1.45*10^{-106}$) for the housekeeping genes and non-housekeeping genes respectively. (**C-W**) Average differential RNA half-life (log2 scale) for bins of increasing differential transcript level and equal numbers of genes, for the X-chromosome (black) and the autosomes (orange), for pair-wise comparisons between the indicated cell lines. The Spearman correlation coefficients and p-values are gathered in Supplementary table S5.

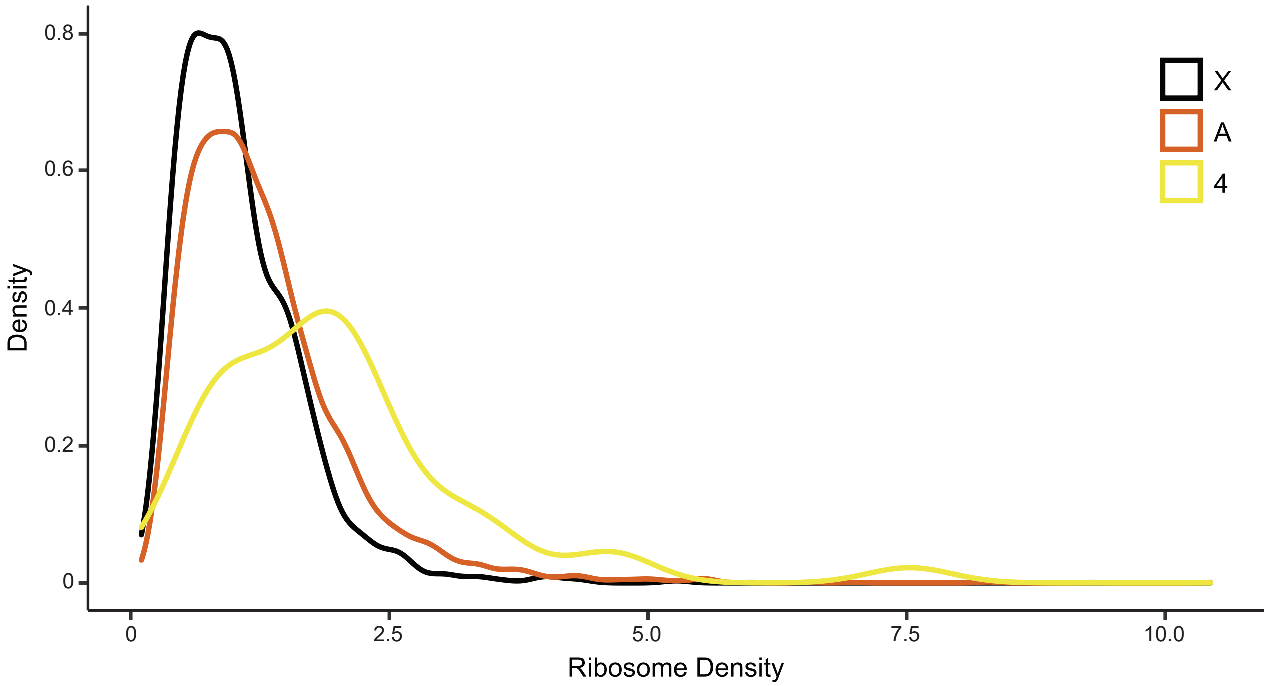

Supplementary figure S5. Distribution of ribosome densities for the X-chromosome (black), the autosomes (orange) and the 4th chromosome (yellow) in S2 cells.

Supplementary Figure S5
